# Possible Mechanisms for Maintenance and Regression of Corpus Luteum Through the Ubiquitin-Proteasome and Autophagy System Regulated by Transcriptional Factors

**DOI:** 10.3389/fendo.2019.00748

**Published:** 2019-11-19

**Authors:** Aamir S. Teeli, Paweł Leszczyński, Narayanan Krishnaswamy, Hidesato Ogawa, Megumi Tsuchiya, Magdalena Śmiech, Dariusz Skarzynski, Hiroaki Taniguchi

**Affiliations:** ^1^Department of Experimental Embryology, The Institute of Genetics and Animal Breeding, Polish Academy of Sciences, Jastrzebiec, Poland; ^2^Indian Veterinary Research Institute, Hebbal Campus, Bengaluru, India; ^3^Graduate School of Frontier Biosciences, Osaka University, Suita, Japan; ^4^Department of Reproductive Immunology and Pathology, Institute of Animal Reproduction and Food Research, Polish Academy of Sciences, Olsztyn, Poland

**Keywords:** ubiquitin-proteasome, autophagy, steroidogenesis, corpus luteum, transcription factors

## Abstract

The corpus luteum (CL) is an important tissue of the female reproductive process which is established through ovulation of the mature follicle. Pulsatile release of prostaglandin F_2α_ from the uterus leads to the regression of luteal cells and restarts the estrous cycle in most non-primate species. The rapid functional regression of the CL, which coincides with decrease of progesterone production, is followed by its structural regression. Although we now have a better understanding of how the CL is triggered to undergo programmed cell death, the precise mechanisms governing CL protein degradation in a very short period of luteolysis remains unknown. In this context, activation of ubiquitin-proteasome pathway (UPP), unfolded protein response (UPR) and autophagy are potential subcellular mechanisms involved. The ubiquitin-proteasome pathway (UPP) maintains tissue homeostasis in the face of both internal and external stressors. The UPP also controls physiological processes in many gonadal cells. Emerging evidence suggests that UPP dysfunction is involved in male and female reproductive tract dysfunction. Autophagy is activated when cells are exposed to different types of stressors such as hypoxia, starvation, and oxidative stress. While emerging evidence points to an important role for the UPP and autophagy in the CL, the key underlying transcriptional mechanisms have not been well-documented. In this review, we propose how CL regression may be governed by the ubiquitin-proteasome and autophagy pathways. We will further consider potential transcription factors which may regulate these events in the CL.

## Introduction

Corpus luteum (CL) formation, an integral part of the female reproductive process, is accomplished through ovulation of the mature follicle. The CL is a transient organ composed of various cells types. These include endothelial cells, immune cells, and luteal cells, which differentiate from follicular cells (granulosa and thecal cells) following ovulation. The CL development is classified as early, mid, late and regression stages in terms of its growth rate, neovascularisation, and rate of progesterone (P_4_) production. In the absence of pregnancy, luteolysis occurs with a decrease in P_4_ synthesis and secretion. Conversely, P_4_ produced by the CL maintains pregnancy in several species. To produce P_4_ in the CL, free cholesterol is transferred to the inner mitochondrial membrane by carrier proteins including steroidogenic acute regulatory protein (STAR). This process involving the P450 cholesterol side-chain cleavage enzyme (p450scc/CYP11A1) converts cholesterol to pregnenolone, the C-21 steroid precursor (1). STAR is a protein that governs the rate-limiting step of gonadal steroidogenesis.

In a non-fertile estrous or menstrual cycle, the CL undergoes luteolysis. In ruminants, pulsatile release of prostaglandin F_2α_ (PGF_2α_) by the uterus leads to regression of luteal cells and renewal of the estrous cycle. The rapid functional regression of the CL, which is characterized by decreased P_4_ production, is followed by structural regression. During the structural regression, luteal cells undergo apoptosis (2–5). Failure of this mechanism is associated with dysfunction of the reproductive cycle and infertility. While we understand the process by which the CL undergoes programmed cell death, the mechanisms governing the degradation of a large quantity of CL proteins over a very short period of time is yet to be elucidated.

The ubiquitin-proteasome pathway (UPP) plays an important role in the degradation of unnecessary proteins. During this process, target proteins are first bound to small ubiquitin proteins and degraded. The UPP is a regulatory mechanism that maintains tissue homeostasis in response to various stressors including oxidative stress. This pathway acts through the endoplasmic reticulum (ER) (6). The UPP governs physiological processes in a variety of gonadal cells (7, 8). Emerging evidence suggests that UPP dysfunction leads to pathology within reproductive system (9). Additionally, the unfolded protein response (UPR) signaling pathway, a cellular stress response associated with the ER, is involved in the development, maintenance, and regression of the bovine CL (10). Female mice lacking Beclin1 (*Becn1*), a regulator of autophagy and the UPR system, display a preterm labor phenotype associated with P_4_ production dysfunction in ovarian granulosa cells (GCs) (11). Interestingly, GRP78, an ER chaperone protein essential for UPR, plays an integral role in the initiation of steroidogenesis through STAR activation at the mitochondrial membrane (12). Moreover, increased oxidative stress in the ER reduces testosterone production in Leydig cells (13). As such, the UPP and UPR are crucial to degrade unnecessary proteins and maintain cellular homeostasis. Unfortunately, the molecular mechanisms governing UPR function in the CL remain poorly understood.

Similar to the UPP system, autophagy degrades unnecessary proteins through the autophagosome. This process is involved in the metabolism of cellular components associated with the UPP under normal and pathologic conditions. The UPP and autophagy systems are closely related mechanisms that remove unnecessary cellar components. They act cooperatively to maintain cellular homeostasis (14). ER stress is an important trigger of the UPP and autophagy. Recent studies have demonstrated that ER stress and autophagy play important roles in structural regression of CL (15, 16). Together, these results suggest that ER stress-mediated autophagy may play an important role in luteolysis.

In view of these findings, we hypothesize that the UPP and autophagy may play important roles in the functional regulation of the CL and luteolysis. In this review, we will explore the molecular mechanisms governing luteal function and regression as well as its interplay with the proteasome-autophagy system.

## Functional Luteal Regression and Transcription Factors Controlling CL Function

**Figure 1:**
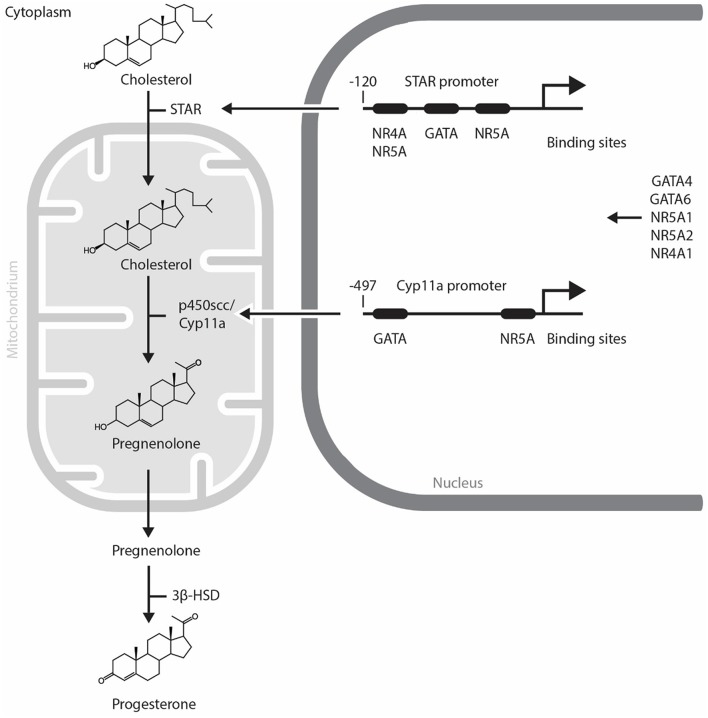
Mechanism of progesterone synthesis and its molecular regulation by GATA and NR5A transcription factors in steroidogenic cells. STAR plays a role in transporting cholesterol to the mitochondria. p450scc converts cholesterol into pregnenolone in the mitochondria. Pregnenolone is finally converted to progesterone by 3β-HSD in steroidogenic cells. STAR and Cyp11a promoters contain evolutionally conserved GATA and NR5A/4A binding sites. GATA, NR4A1, and NR5A transcription factors control the expression of steroidogenic enzymes including STAR, p450scc/Cyp11a. NR5A1, Nuclear Receptor Subfamily 5 Group A Member 1; NR5A2, Nuclear Receptor Subfamily 5 Group A Member 2; NR4A1, Nuclear Receptor Subfamily 4 Group A Member 1; p450scc, cytochrome p450 side-chain cleavage enzyme; STAR, Steroidogenic Acute Regulatory Protein; 3β-HSD, 3β-hydroxysteroid dehydrogenase.

Progesterone, the major hormone of CL is elaborated by small and large luteal cells, that are derived from the follicular theca interna and granulosa cells, respectively. The non-steroidogenic component of CL comprises of endothelial cells, pericytes, fibroblasts and immune cells. Small luteal cells are stimulated by luteinizing hormone (LH) which is secreted by the pituitary gland. P_4_ secreted by large luteal cells represents the basal P_4_ level. The P_4_ level derived from small luteal cells is known as LH-induced P_4_. The LH receptor is a G protein-coupled receptor with seven membrane spanning domains (17). Once LH binds to its receptor, the second messenger cyclic AMP is released and protein kinase A (PKA) is subsequently activated. PKA phosphorylates various proteins and in turn modulates their function (18). Thereafter, transcription factors induce the expression of steroidogenic enzymes including *STAR, Cyp11a*, and 3β-hydroxysteroid dehydrogenase (3β-HSD). STAR plays a role in transporting cholesterol to the mitochondria. P450scc converts cholesterol into pregnenolone in the mitochondria. Pregnenolone is finally converted to P_4_ by 3β-HSD in the luteal cells [(19), Figure 1].

The regulation of steroidogenesis in the CL involves the temporal expression of genes coding for a variety of steroidogenic enzymes. As the rapid upregulation of steroidogenic enzyme gene expression is required, it is likely that the acute regulation of steroidogenesis in the CL is regulated by transcription factors (Figure 1) and change in the expression and activity of these transcription factors trigger functional CL regression. In this regard, NR5A1 (Nuclear Receptor Subfamily 5 Group A Member 1, also known also as adrenal 4 binding protein/steroidogenic factor 1: Ad4BP/SF-1), a regulator of multiple P450 hydroxylases and other components of the steroidogenic program, was first isolated from the adrenal gland (20, 21). Since then, several researchers have identified other transcription factors that regulate the promoter activity of *Star, CYP11A1, HSD3B2*, and *CYP17* [reviewed in (22)]. NR5A1 is abundant in the CL during the midluteal phase and binds to the *Star* promoter *in vivo* (23). Moreover, NR5A1 regulates *Star* and *Cyp11a* gene expressions in luteal cells and the CL of many species (23–26). A luteal cell-specific *Nr5a1* knockout (KO) has not been reported. The ovaries of a newborn mice lacking Nr5a1 in GCs are comparable with wild type; however, Nr5a1-deficient adult females lack CL and suffer from sterility (27).

NR5A2 (also known as Fetoprotein transcription factor: FTF, liver receptor homolog 1: LRH-1), another NR5A family member, is also present in the ovary (28). Interestingly, NR5A2 recognizes the same consensus binding sequence as NR5A1 and may regulate similar steroidogenic enzyme target genes. NR5A2 is the most prominent NR5A factor in the CL (29). Similar to NR5A1, NR5A2 is a potent regulator of steroidogenic gene expression in the CL (23, 29). Luteal specific KO of *Nr5a2* is linked with luteal insufficiency, which suggests that this factor plays a crucial role in luteal formation and function (30). Additionally, *Nr5a1* and *Nr5a2* KO mice individually exhibit luteal disruption with downregulated steroidogenic enzyme gene expression (functional luteal disruption) and severe structural damage (27, 29, 31). Both these factors, therefore, play prominent roles in luteal function, development, and regression.

While the role of NR4A1 (Nuclear Receptor Subfamily 4 Group A Member 1) in steroidogenesis in the CL is yet to be elucidated, its expression is upregulated by cAMP, a second messenger of several pituitary hormones including LH. NR4A1 is present in the theca cells, GCs, and luteal cells in the human ovary (32, 33). Moreover, NR4A1 is known to regulate *StAR* gene expression activity in mouse Leydig cells (34, 35). On the other hand, NR4A1 levels are upregulated by PGF_2α_ in pseudopregnant rats (36). Further studies are needed to elucidate the control of NR4A1 levels and its role in luteal steroidogenesis.

**Figure 2:**
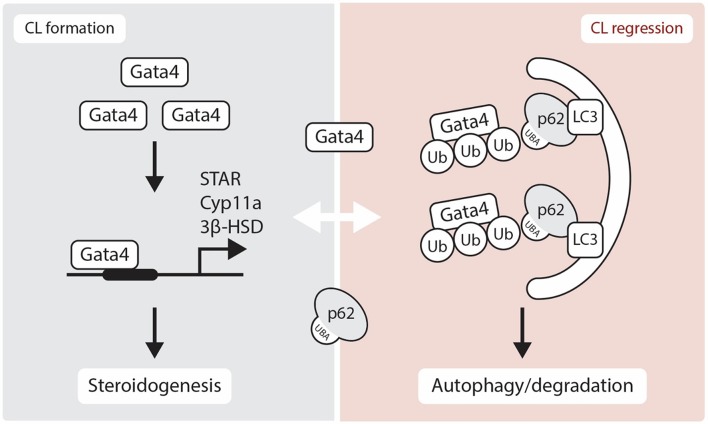
Schematic representation showing the role of GATA4 in luteogenesis and luteolysis. Gata4 is located in the nucleus and regulates several steroidogenic enzymes gene expression. Although p62 is thought to be localized in the cytoplasm, recent studies have suggested that p62 physically interacts with nuclear proteins. Here, we show a hypothetical regulation of GATA4 transcription factor through an interaction with p62 in the luteolytic CL. STAR, Steroidogenic Acute Regulatory Protein; 3β-HSD, 3β-hydroxysteroid dehydrogenase; CYP11A, Cytochrome P450 Family 11 Subfamily A Member; Ub, Ubiquitin; LC3, Microtubule-associated protein 1A/1B-light chain 3; UBA, Ubiquitin-Associated domain.

The gonads also express several GATA factors that are known to regulate steroidogenic gene expression [reviewed in (37)]. In the CL of *Gata4* and *Gata6* conditional double knockdown mice, a reduction in P_4_ production is observed along with an acute inhibition of expression of genes in the steroidogenic pathway including *Star, Hsd3b1*, and *Cyp11a* [(38), Figure 2]. Moreover, *GATA4* and *GATA6* mRNA and protein were identified in bovine CL and it is suggested that GATA6 may be involved in the regulation of *STAR* expression in this species (23). As GATA4 and GATA6 are proposed to physically and functionally interact with NR5A1 and NR5A2 to upregulate steroidogenic enzyme gene transcription via the *HSD3B2* and *Cyp19a* promoter (39), one can expect that they also play important roles in steroidogenesis in the CL. While we have good insights into how steroidogenesis is likely turned on by transcriptional factors in the CL, the mechanism governing its inhibition during functional luteolysis remains obscure. NR0B1 inhibits transcriptional cooperation between GATA4 and NR5A1 in testicular cells, suggesting that it might possibly mediate the inhibitory effect of PGF_2α_ on P_4_ in the CL (40, 41).

## Structural CL Regression and Apoptosis

Reduced P_4_ secretion begins during the late luteal phase and leads to CL structural regression. This structural regression occurs through apoptosis which involves nuclear fragmentation (3, 4) as well as caspase 3 and p53 activation (42–44). Detailed information of apoptosis-mediated CL regression is well-reviewed in (45). Our group has identified that apoptosis in bovine CL is induced by the interaction between cytokines and Fas/FasL (46). It is well-established that PGF_2α_ triggers apoptosis during luteolysis. Immune cells and cytokines play important roles in structural luteolysis as evidenced by increased T-lymphocyte and macrophage influx during CL regression (47). In bovine luteal cells, Fas-FasL mediated cell death plays a crucial role in luteal cell apoptosis. This process is induced by interferon gamma (IFNγ) and tumor necrosis factor alpha (TNFα) plays a stimulatory role. Treatment of luteal cells with Fas ligand, in presence of IFNγ and TNFα, leads to the formation of apoptotic bodies, which supports the notion that these cytokines are implicated in luteal regression (46). Moreover, IFNγ and TNFα induce mouse luteal cell apoptosis (48). Macrophages degrade extracellular matrix (ECM) and phagocytize degenerated luteal cells leading to the release of cytokines including TNFα, interleukin-1β (IL-1), and IFNγ (49). The intraluteal TNFα level increases significantly during both spontaneous and induced *in vivo* luteolysis in microdialyzed CL (50). It is, therefore, likely that TNFα stimulates synthesis of luteal PGF_2α_. This modulation of TNFα levels also leads to increased nitrates/nitrites, and stabilization of nitric oxide metabolites (51). TNFα acts in concert with IFNγ to induce luteolysis (46, 52). Hojo et al. (53) have demonstrated that necroptosis is involved in structural regression of CL due to receptor-interacting serine/threonine-protein kinase (RIPK)1 and 3 induction following the treatment of the luteal cells with the inflammatory cytokines IFNγ and TNFα in bovine CL (53). Increased RIPK1 and 3 protein expression is also found in PGF_2α_-induced CL regression, suggesting necroptosis is involved in CL regression. Administration of PGF_2α_ in livestock with functional CL induces luteal regression.

Following functional and structural regression of the CL, proteins in the CL are degraded and removed by regulatory mechanisms. We describe hereafter the proteasome-ubiquitin system and the autophagy mechanisms involved in protein degradation and removal of unnecessary tissue structures.

## Proteasome and CL Regulation

**Figure 3:**
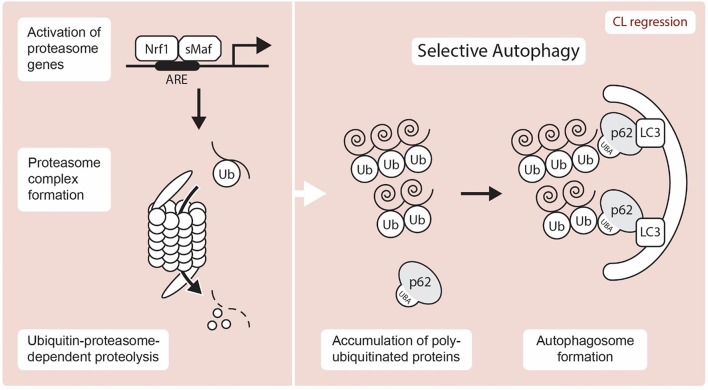
Schematic representation showing UPP and autophagy mediated proteolysis. A transcription factor, Nrf1 is liberated from the ER after stress and translocates to the nucleus, where it induces the expression of proteasome subunit genes through the ARE (antioxidant response element) by hetero-dimerizing with a small Maf (sMaf) protein. Selective autophagy is known to exert proteolysis through the recognition of unnecessary target protein via p62-LC3-autophagosome. Nrf1, NF-E2-related factor 1; sMAF, small musculoaponeurotic fibrosarcoma proteins; ARE, antioxidant response element; Ub, Ubiquitin; LC3, Microtubule-associated protein 1A/1B-light chain 3; UBA, Ubiquitin-Associated domain.

The UPP plays major roles in the degradation of unnecessary proteins. The targeted proteins are bound by small ubiquitin proteins (Figure 3). The role of ubiquitin is tightly regulated by several enzymes namely ubiquitin-activating enzyme (E1), ubiquitin-conjugating enzyme (E2), and ubiquitin ligase (E3) (54). On the other hand, the ubiquitinated proteins are degraded by a huge protein complex called the 26S proteasome. The 26S proteasome complex consists of two subclass complexes: the 19S and 20S particles. It is known that the UPP plays important role in the reproductive system (7, 8). Emerging evidence also suggests that dysfunction of the UPS leads to multiple diseases, including the dysfunction of the male and female reproductive tracts (9, 55).

Surprisingly, the UPP system in the CL is not well-characterized and only a very limited number of proteasome genes have been identified in the CL. Nonetheless, the proteasome plays a central role in the degradation of unnecessary proteins, which are labeled with ubiquitin proteins. Since the mammalian CL is renewed after each infertile estrous/menstrual cycle, understanding how its proteins are degraded is an important question that remains unanswered. The proteasome inhibitors MG115 and MG132 reduced both mRNA and protein expression of StAR in the rat adrenal cortex (56). On the other hand, the stability of breast cancer susceptibility gene 1 (*BRCA1*) and its partner BRCA1-associated RING domain protein 1 (BARD1) is regulated by proteasome degradation in human ovarian GCs (57). This regulatory process is also associated with both cAMP-dependent and cAMP-independent steroidogenic processes. Forskolin-induced *Cyp19a* expression is blocked by MG132 treatment, which suggests that proteasome inhibition downregulates steroidogenesis. It appears that the proteasome-ubiquitin system plays an important role in steroidogenic gene expression and therefore further functional studies about the roles of UPP in luteal steroidogenesis are required. At the molecular level, NF-E2-related factor 1 (NRF1), a transcription factor involved in the stress response, has garnered significant attention due to its implication in protein clearance via the UPP (Figure 3). NRF1 is a member of the cap “n” collar (CNC)-bZip transcription factor family and is localized at the ER until an external stressor is present. Upon activation by an external stressor, NRF1 is liberated from the ER and translocates to the nucleus, where it stimulates the expression of proteasome subunit genes via the antioxidant response element (ARE) by hetero-dimerizing with a sMAF (small musculoaponeurotic fibrosarcoma) proteins and to maintain protein homeostasis [(58, 59); Figure 3]. Proteasome and deubiquitination enzyme gene expressions are regulated by NRF1 (60, 61). A recent study has demonstrated that deletion of *Nrf1* causes downregulation of several proteasome genes with the accumulation of ubiquitinated proteins and p62/SQSTM1, an autophagy marker (61). Much like ubiquitination, deubiquitination also contributes to proteostasis by regulating cellular levels of free monomeric ubiquitin. However, while Proteasome subunit beta type (*PSMB*) 8 and 9 gene expression, has been identified in the bovine CL (62), the roles of the UPP and its regulation by NRF1 require further study.

## UPR and CL Regression

**Figure 4:**
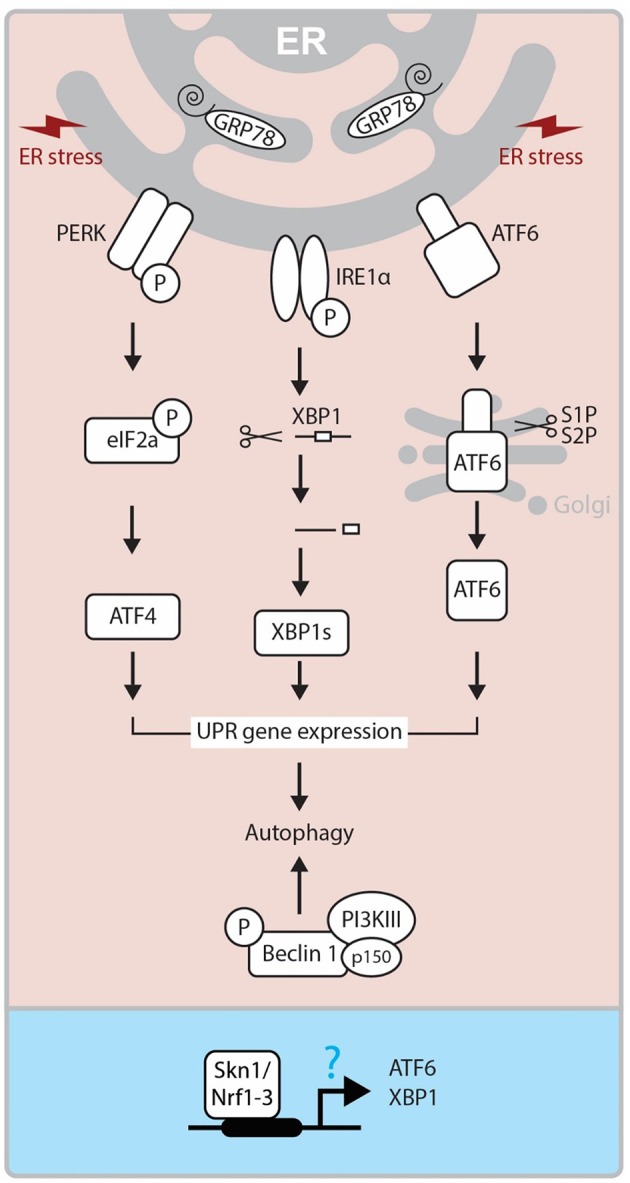
Molecular mechanism of UPR. ER Stress-activated three pathways (IRE, PERK, and ATF6) control the activity of XBP1s, ATF4, ATF6 transcription factors and regulate UPR related gene expressions, which triggers autophagy. On the other hand, Beclin1 forms a complex with PI3KIII and p150, and this also triggers autophagy mechanism. In *Caenorhabditis elegans*, SKN-1, the ortholog of human Nrf1-3, has been reported to regulate ATF6 and XBP1 expressions at the promoter level. ER, endoplasmic reticulum; GRP78, Glucose-regulated protein; PERK, PKR-like eukaryotic initiation factor 2α (eIF2α) kinase; IRE1, inositol-requiring transmembrane kinase/endoribonuclease 1; ATF6, activating transcription factor-6; XBP1, X-box-binding protein 1; XBP1s, spliced XBP1 protein; S1P and S2P, site-1 and site-2 proteases; PI3KIII, Class III PI 3-kinase; Skn1, Protein skinhead-1.

Another pathway that has been implicated in CL regression is ER stress pathway (10, 15, 63). The ER stress is a cellular phenomenon induced by diverse stimuli disturbing the protein folding in the ER (64, 65). In response to ER stress, UPR pathway is activated to restore the ER homeostasis. The UPR pathway involves the actions of three signaling proteins: protein kinase RNA-like ER kinase (PERK), inositol-requiring enzyme-1/X-box-binding protein (IRE1/XBP-1), and activating transcription factor 6 (ATF6) (66, 67). The PERK and ATF6 are normally in inactive form due to their association with BiP (Binding immunoglobulin Protein; also known as Glucose-regulated protein-Grp78), an ER resident chaperone (Figure 4). The main role of the CL is to secrete P_4_, which is essential for maintaining pregnancy. While the steroidogenic process diverges into several separate pathways which lead to the synthesis of different steroid products, StAR is significantly involved in this process and regulates the rate-limiting step in P_4_ production in the CL. Reduced expression of GRP78, a ER chaperone protein critical for UPR, cause inhibition of StAR protein expression and activity in steroidogenic cells (12). Moreover, female mice lacking Becn1, a regulator of autophagy and the UPR system, have a defect in P_4_ production in the ovarian GCs and display a preterm labor phenotype (11). These results suggest that the UPR system may regulate P_4_ production through various mechanisms. On the other hand, Skn1, an ortholog of Nrf1-3 in *C. elegans*, regulates UPR signaling and transcription factor genes. SKN-1 contributes to the expression of core UPR factors, *pek-1* and *atf-6* (68). Loss of Skn1 inhibits upregulation of Xbp1 gene expression by ER-stress. Chromatin immunoprecipitation studies indicate that endogenous SKN-1 accumulates at the *xbp-1* site of transcription in the presence of ER stress (Figure 4). In the steroidogenic cells, intracellular cholesterol is stored in an esterified form. In response to trophic hormone signals coming from the pituitary (e.g., LH, ACTH), cholesterol esterase hydrolyzes cholesterol ester into free cholesterol in target steroidogenic cells. In luteal cells, cholesterol is converted into different types of steroids including P_4_ by several steroidogenic enzymes. The gene expression of these enzymes is tightly regulated by several transcription factors. Interestingly, a recent study has revealed that Nrf1 controls cholesterol homeostasis by binding to cholesterol (69). Thus, it is important to determine whether Nrf1 controls steroidogenesis via UPP and UPR modulation in the CL.

As an ER transmembrane protein, IRE1 is essential for UPR and is only activated through binding to unfolded proteins (70). Under the conditions of severe and chronic ER stress, the UPR pathway is not able to cope and cellular dysfunction or death ensues through the activation of both the extrinsic and intrinsic apoptosis pathways (71). Apoptosis is initiated by PERK/eIF2α mediated induction of proapoptotic TF CHOP (Transcriptional factor C/EBP homologous protein), and IRE1 dependent activation of TRAF2 (TNF receptor-associated factor 2). This process stimulates the c-Jun NH2-terminal kinase (JNK) pathway (64, 72). The role of ER stress-mediated apoptosis is well-established in many reproductive events, including follicular atresia (73) and CL regression (10, 15, 63). Yang et al. (15) demonstrated that ER stress markers like Grp78, CHOP, ATF6α and caspase 12 are significantly expressed at the mRNA and protein levels in the late luteal stage during spontaneous and PGF_2α_ induced luteolysis (15). These findings were further supported by decreased expression of ER stress markers and apoptosis in luteal cells treated with tauroursodeoxycholic acid, which functions as a chemical chaperone and reduces ER stress (15, 63). Thus, it is plausible that ER stress-mediated UPR controls both functional and structural luteal regression although further studies are needed to elucidate its molecular mechanism.

## Autophagy and CL Regression

Redundant cellular components are recognized and transferred to the lysosome by a mechanism known as autophagy. In autophagy, mitophagy selectively degrades mitochondria and pexophagy selectively degrade peroxisomes. Selective autophagy also plays major roles in the degradation of cellular components such as aggregated proteins (74, 75). To recognize target components and properly bring them to the lysosome, an adapter protein involved in autophagy requires at least two domains. One region binds to the target protein and the other domain is necessary for transporting the target to an autophagy mechanism. Autophagy receptor p62 (also known as sequestosome-1) is an adaptor protein which mediates the interaction between selected proteins and autophagosomes. The receptor p62 possesses a LC3-Interacting Region (LIR) motif that interacts with LC3. This interaction allows the receptor p62 to bind to the autophagosomal membrane (Figure 3). p62 co-aggregates with the target at the phagophore due to its homopolymerisation property mediated by its PB1(Phox/Bem1p) domain. Interaction of p62 with Atg8/LC3 on the autophagosomal membrane is extremely important for transport. An increase in the expression of LC3-II relative to the expression of LC3-I occurs during autophagy. Although studies on autophagy in the mammalian CL are limited, the link between autophagy and apoptosis is well-established (76, 77). In fact, expression of various autophagy related factors is increased in the late luteal and regression stages of the CL. Increased expression of beclin protein during the late luteal stage in the sow suggests a role in the removal of unwanted proteins during luteolysis (78). LC3-II, an autophagy marker is expressed more in the late than middle stage CL in cattle (42). Similar results were obtained in rat CL as well (16). These findings suggest that autophagy is highly involved in luteal regression in several species. Lipid droplets are unique organelles in the luteal cells enriched with cholesterol esters, that serve as precursor for steroidogenesis. Inhibition of autophagy in luteal cells via deletion of *Becn1* causes failure of lipid droplet formation, and leads to reduced P_4_ secretion, demonstrating the critical role of Becn1/ autophagy in luteal steroidogenesis (11). Therefore, the relationship between steroid synthesis and autophagy in the CL should be studied. The p62 receptor exerts its physiological functions including signal transduction regulation, intracellular protein localization (trafficking), and selective autophagy of ubiquitinated proteins through its interaction with various proteins (79). The p62 receptor binds to ubiquitin containing aberrantly aggregated proteins. Since p62 has a ubiquitin associated (UBA) domain and binds to ubiquitinated proteins, p62 plays an important role in selective autophagy. Presently, LC3 and p62 are widely used as autophagy markers. Interestingly, recent studies have revealed that the p62 receptor, a primarily cytoplasmic protein, plays an important role in the nucleus by interacting with several nuclear factors (described below, Table 1) that are expressed in the CL (23, 36, 38).

**Table 1:** p62/SQSTM1 interacting nuclear proteins in the regulation of autophagy.

**Protein**	**Biological Process**	**References**
GATA4	GATA family of zinc-finger transcription factors	(80, 81)
ARIP4	AR interacting protein 4-a Rad54 family member and a SNF2 chromatin remodeling factor	(82)
TP53INP1	Tumor Protein P53 Inducible Nuclear Protein-Tumor suppressor/Autophagy	(83)
Rad51	RAD51 Recombinase-DNA damage repair	(84)
PARP-1	Poly (ADP-ribose) polymerase 1-DNA repair	(85)
LC3	Microtubule-associated protein 1A/1B-light chain 3-Autophagy	(86)
AR	Androgen receptor	(87)
PPARα	Peroxisome proliferator-activated receptor alpha	(88)
NR4A1/Nur77	Nerve growth factor IB(NGFIB)/Nur77 /NR4A1-Transcriptional Factor	(89)

## Possible Transcriptional Regulation of Autophagy Through the Autophagy Receptor p62/SQSTM1 in the CL

The nuclear transcription factor GATA4 binds to p62 and is degraded through autophagy [(90), Figure 2]. The lysosomal autophagic pathway regulates GATA4 during ionizing radiation-induced and progerin-induced senescence in human mesenchymal stem cells (hMSCs) and fibroblasts, respectively (80, 81). In both fibroblast and hMSCs senescent cells, GATA4 accumulates as a consequence of the loss of physical interaction between p62 and GATA4 (80, 81). While the various functions of GATA factors in luteal P_4_ regulation are well-characterized, their role in autophagy is yet to be elucidated (Figure 2). Accordingly, SKN-1/Nrf1-3 and ELT-2/GATA transcription factors may regulate the expression of proteasome subunit genes as well as oxidative and heat-stress response genes (91). As NRF family members play prominent roles in the proteasome-autophagy system (61, 92), the relationship between GATA factors and NRF family members in the CL merits further investigation.

Androgen receptor-interacting protein 4 (ARIP4) interacts with sumoylated nuclear receptors such as NR5A1, NR5A2, NR3C1 (GR: glucocorticoid receptor) and NR3C4 (AR: androgen receptor) (93). Multiple studies have shown that steroidogenic genes are regulated by NR5A1. This occurs through direct interaction with transcription factors such as GATA4 (39, 94). The UBA domain of p62 interacts with a novel domain in ARIP4, named SQSTM1/p62 interaction Domain (SID). This domain possesses binding properties similar to ubiquitin. The p62 receptor negatively regulates ARIP4 levels under starvation induced autophagy. This indicates that the interaction between ARIP4 and p62 is involved in the regulation of ARIP4 protein levels during autophagy (82). Considering the dual role of ARIP4 in steroidogenic gene regulation via NR5A1 and autophagy via p62, it will be interesting to further investigate its role during active steroidogenesis and autophagy-mediated CL regression (95, 96).

NR4A, a member of the nuclear receptor superfamily, plays an important role in a variety of cellular processes (97). NR4A1 functions as a nuclear transcriptional factor and activates steroidogenic gene expression in gonadal cells (32, 98). Hu et al. (89) first showed that NR4A1 physically interacts with p62 and accumulates in the mitochondria when mouse embryonic fibroblasts (MEFs) are treated with TNFα and celastrol (89). This suggests that TNFα-mediated apoptosis in the CL may be controlled by NR4A1-dependent regulation of mitochondrial autophagy. Further studies are needed to investigate autophagic regulation of nuclear receptors by p62 in the CL.

TP53 INP1 (tumor protein 53-inducible nuclear protein 1) is a tumor suppressor whose expression is reduced in various cancers. TP53 INP1-LC3 binding occurs via its functional LC3 interaction region (LIR). When TP53 INP1 is highly expressed, TP53 INP1-LC3 interaction is stronger than the p62-LC3 interaction. This inhibits the binding of LC3 to p62 and in turn enhances p62-mediated protein degradation (83). TP53 INP1 also induces autophagy-dependent cell death (83). Since P53 protein is an apoptotic factor in the CL (43), functional analysis of TP53 INP1 in the CL is needed.

PARP-1 [Poly (ADP-ribose) polymerase 1], a key factor in DNA repair, is a partner protein of p62 (85). PARP-1 also binds to LC3 and phosphorylated Unc-51 like autophagy activating kinase 1(ULK1), which are both key factors in autophagy. Rad51 plays a central role in DNA double strand break (DSB) repair through homologous recombination (HR) and also interacts with p62 (84). As autophagy occurs primarily in the cytoplasm, elucidating the role of crosstalk between nuclear localized proteins and autophagy signaling is crucial.

It is worth noting that p62 regulates the binding between Nrf2 and Keap1 (Kelch ECH associating protein 1) although they interact in the nucleus (99). The Nrf2-Keap1 interaction is known to be one of the cellular mechanisms to protect the cells from oxidative stresses. To regulate this mechanism, Nrf2 transcription factor is continuously degraded when its partner protein Keap1 binds. In canonical pathway, the binding of Keap1 controls Nrf2 transcriptional activity (100). However, in non-canonical pathway, p62 binds to Keap1, and this interaction induces a selective autophagy pathway and prevent the interaction between Keap1 and Nrf2 (99). Consequently, Nrf2 transcriptional activity is enhanced and this acts as stress sensor mechanism. To our surprise, many factors identified as nuclear partners of p62 are transcription factors involved in gene regulation of steroidogenic enzymes. Thus, in the near future, the relationship between luteal functional regulation and autophagy-controlled luteal regression will be clarified and we will have better understanding of how luteal function (P_4_ production) and regression (apoptosis/autophagy) are orchestrated.

## Conclusion

Understanding the intracellular homeostatic mechanisms during the maintenance and lysis of CL will pave way for addressing infertility due to luteal dysfunction. Previous research has suggested that dysfunction of the ubiquitin-proteasome and autophagy systems leads to many disorders, including diseases of the male and female reproductive system. However, its molecular basis is not well-studied. We hope that transcriptional regulation of proteasome and autophagy systems during luteolysis will be unraveled in the near future. Elucidation of the transcription factor-proteasome/autophagy axis also could enable efficient recovery from stress situations which will lead to a significant advancement in the field of animal reproduction.

## Author Contributions

AT and HT conceived, coordinated, wrote and edited the manuscript. AT, NK, HO, and MT wrote, interpreted data, and revised the manuscript. PL, MŚ, and DS edited and revised the manuscript.

### Conflict of Interest

The authors declare that the research was conducted in the absence of any commercial or financial relationships that could be construed as a potential conflict of interest.
